# Inherent and unpredictable bias in multi-component DESPOT myelin water fraction estimation

**DOI:** 10.1016/j.neuroimage.2019.03.049

**Published:** 2019-03-28

**Authors:** Daniel J. West, Rui P.A.G. Teixeira, Tobias C. Wood, Joseph V. Hajnal, Jacques-Donald Tournier, Shaihan J. Malik

**Affiliations:** a Department of Biomedical Engineering, School of Biomedical Engineering and Imaging Sciences, King's College London, St. Thomas' Hospital, London, SE1 7EH, United Kingdom; b Centre for the Developing Brain, School of Biomedical Engineering and Imaging Sciences, King's College London, St. Thomas' Hospital, London, SE1 7EH, United Kingdom; c Institute of Psychiatry, Psychology & Neuroscience, King's College London, 16 De Crespigny Park, Camberwell, London, SE5 8AB, United Kingdom

**Keywords:** mcDESPOT, Myelin water fraction, Stochastic region contraction, Intercompartmental exchange, White matter-grey matter contrast, Quantitative MRI

## Abstract

Multicomponent driven equilibrium steady-state observation of T_1_ and T_2_ (mcDESPOT) aims to quantify the Myelin Water Fraction (MWF) using a two-pool microstructural model. The MWF has been used to track neurodevelopment and neurodegeneration and has been histologically correlated to myelin content. mcDESPOT has a clinically feasible acquisition time and high signal-to-noise ratio (SNR) relative to other MWF techniques. However, disagreement exists in the literature between experimental studies that show MWF maps with plausible grey matter-white matter (GM-WM) contrast and theoretical work that questions the accuracy and precision of mcDESPOT. We demonstrate that mcDESPOT parameter estimation is inaccurate and imprecise if intercompartmental exchange is included in the microstructural model, but that significant bias results if exchange is neglected. The source of apparent MWF contrast is likely due to the complex convergence behaviour of the Stochastic Region Contraction (SRC) method commonly used to fit the mcDESPOT model. mcDESPOT-derived parameter estimates are hence not directly relatable to the underlying microstructural model and are only comparable to others using similar acquisition schemes and fitting constraints.

## 1 Introduction

Originally proposed by Deoni et al. (2008), “multicomponent driven equilibrium steady-state observation of T_1_ and T_2_” (mcDESPOT) is the multi-component adaptation of the established driven-equilibrium single-pulse observation of T_1_ (DESPOT1), also called the Variable Flip Angle (VFA) method, and T_2_ (DESPOT2) techniques that enable accurate and precise determination of longitudinal and transverse relaxation times respectively (Deoni et al., 2003). mcDESPOT attempts to quantify the proportion of MR-visible water protons in a voxel that are trapped between myelin lipid bilayers, referred to as the Myelin Water Fraction (MWF). The remaining MR-visible protons are contained in the intra- or extra-cellular (IE) spaces (Does, 2018). As myelin water is a substantial component of myelin composition, the MWF has been suggested as a direct measure of myelin content through histological studies, and hence has potential as a biomarker for neurological conditions (Laule et al., 2006; Wood et al., 2016).

Multiexponential T_2_ (MET_2_) imaging remains the gold-standard for myelin water imaging but is hindered by a long acquisition time and limited coverage (MacKay et al., 1994; Alonso-Ortiz et al., 2015). Instead, mcDESPOT is based on rapid spoiled gradient-recalled (SPGR) echo and balanced steady-state free precession (bSSFP) sequences, and hence benefits from high SNR efficiency and clinically feasible scan times with whole-brain coverage and reasonable isotropic resolution. Signals are fitted to a tissue model comprising fast-relaxing (myelin water) and slow-relaxing IE pools of magnetisation. The model can be described by the relaxation times of both pools: *T_1F_*, *T_1S_*, *T_2F_* and *T_2S_* where the subscripts *F* and *S* refer to fast and slow respectively; their relative sizes, represented by equilibrium magnetisations *M_0F_* and *M_0S_*; and the intercompartmental exchange rates, *k_FS_* and *k_SF_*. Under the assumption that *M_0F_*+*M_0S_* = *1*, *M_0F_* is the myelin water fraction.

It is accepted that the MWF derived from mcDESPOT is overestimated compared to alternative methods (Alonso-Ortiz et al., 2015; Deoni et al., 2013). Deoni et al. proposed that this could be due to an ill-conditioned fitting approach, ill-posed tissue model, and/or neglect of magnetisation transfer (MT) effects (Deoni and Kolind, 2015). Lankford and Does (2013) computed Cramer Rao Lower Bounds to conclude that mcDESPOT using “feasibly attainable signal-to-noise ratios cannot provide parameter estimates with useful levels of precision”. Yet, using the Stochastic Region Contraction (SRC) (Berger and Silverman, 1991) fitting method, a range of studies have produced plausible MWF maps that show reasonable grey matter-white matter (GM-WM) contrast (Zhang et al., 2015a; Deoni, 2011) and can produce realistic developmental myelination trajectories (Deoni et al., 2012, 2013). As also suggested by Lankford and Does, there are two possible explanations for this discrepancy: (i) the model does not satisfactorily describe the tissue signal response or (ii) the estimation process is biased (their analysis assumed an unbiased estimator).

On the first point, MT is the most obvious candidate to explain such a discrepancy. On-resonance MT effects can result in significant deviations from signal models based purely on the Bloch equation, and are particularly relevant for the short repetition time pulsed sequences used by mcDESPOT. For example MT-effects can attenuate the bSSFP signal by over 30% in brain tissue (Bieri and Scheffler, 2006). This is an issue for VFA measurement because each flip angle is realised by scaling the amplitude or duration of the RF pulse, which causes the energy per pulse and hence the saturation of the macromolecular pool to be dependent on flip angle. It has been shown that this can cause systematic errors of approximately 10% in DESPOT1 measurements (Ou and Gochberg, 2008). Zhang et al. (2015b) found large differences in estimated mcDESPOT parameters depending on the pulse duration, although the effect on MWF itself was small. Their explanation was that this may be due to the complexity of the search space involved in model fitting. Liu et al. (2016) attempted to account for this by proposing a two-stage fitting process that considers macromolecular pool magnetisation in addition to the IE and myelin water. They show that this leads to a reduction in estimation bias, at the cost of estimating more parameters. Teixeira et al. proposed to mitigate the effect of variable saturation power in VFA methods by using novel RF pulse types that change the flip angle while keeping the saturation power fixed (Teixeira et al., 2018). Recently, we demonstrated that this approach would also work for mcDESPOT, but that the expected values of the measured parameters (including MWF) would be modified depending on the amount of applied RF power (West et al., 2018).

On the estimation process itself, the SRC method is a likely source of bias given that the realised parameter variance is much smaller than would be expected from an unbiased estimate (Lankford and Does, 2013). As an alternative, Bouhrara et al. (Bouhrara and Spencer, 2016, 2017) developed a Bayesian Monte Carlo (BMC) fitting approach that provides improved parameter estimation accuracy and precision compared to SRC but requires computationally intensive high dimensional integration for parameter marginalisation and yields more variable parameter estimates compared to the literature. This method assumed no exchange between water pools in order to simplify the estimation. However, there is abundant evidence in the literature (Dortch et al., 2013; Harkins et al., 2012) that intercompartmental exchange does exist in biological tissue. Recent work by van Gelderen and Duyn used a more realistic multilayer model of exchange within the myelin sheath and derived exchange rates notably faster than previous estimates, implying that exchange is an important part of tissue microstructure that cannot be neglected (van Gelderen and Duyn, 2018).

In this article, we present a study of the stability and reproducibility of parameter estimation in mcDESPOT using SRC fitting both with and without exchange, addressing two central questions: (i) how are apparently biologically plausible measurements made from mcDESPOT with relatively high precision? and (ii) what are the estimation biases that result from the ‘standard’ SRC fitting approach?

## 2 Materials and methods

Our methodology comprises both simulated and in-vivo investigations. Simulation work focused on the model fitting aspects by considering a best-case scenario where the model perfectly describes the tissue response. The presence/absence of intercompartmental exchange in both the simulated data and the fitted model was also investigated. For the in-vivo study, we employed the Constant Saturation Magnetisation Transfer (CSMT) method to minimise confounding MT effects (Teixeira et al., 2017).

SRC was used for model fitting, and was implemented as specified in Deoni et al. (Deoni, 2011) In this method, a large number of random combinations of model parameter values are chosen from within an initial bound set, and then the corresponding signals and sum-of-squares residual against the data computed for each. The best (lowest residual) 50 combinations are then used to redefine the bounds to gradually contract the search space; an ‘expansion factor’ prevents over-contraction. This procedure is iterated until either the maximum number of iterations is reached, or the upper and lower bounds are within some small tolerance of one another. Following Bouhrara et al. we used 40,000 random candidates at each iteration, maximum 30 iterations, and a tolerance of 1% for convergence - this is more computationally expensive than other implementations, but has been shown to better ensure convergence (Bouhrara et al., 2016). Simulations were implemented in MATLAB 2018a with signal functions coded in C++/MEX using the Eigen linear algebra library (Guennebaud and Jacob, 2010). We chose to use a two-pool model without any ‘semisolid’ pool that would interact via magnetisation transfer. The signals were hence modelled using Equations (1)–(7) from Deoni et al. (2008). SPGR and bSSFP data were normalised to the mean signal from each sequence (Deoni et al., 2013). According to UK research councils’ Common Principles on Data Policy, all simulation code supporting this study will be openly available at: http://doi.org/10.5281/zenodo.2613725.

### 2.1 Simulated tissue models and acquisitions

Different initial bound sets and acquisition schemes from the literature are summarised in Table 1 and Table 2 respectively. Simulated data was generated for different sets of tissue parameters, shown in Table 3. Here ‘HB’ is based on parameters used by Bouhrara et al. obtained from “human brain imaging” (Bouhrara et al., 2016); WML is ‘white matter--like’ (shorter T_1_, T_2_ and larger MWF); GML is ‘grey matter-like’ (longer T_1_, T_2_ and smaller MWF) and INT has intermediate properties.

### 2.2 Signal model and search space behaviour

mcDESPOT is characterised by a complex, six-dimensional search space (seven if off-resonance is also estimated, which we exclude in this work). In order to visualise the search space, we examined the normalised root mean-square residuals, *η*, defined as:

(1) $$\eta (\theta ,\hat{\theta })=\frac{\sqrt{\frac{1}{N}\sum_{j=1}^{N}{\left(S_{j}(\theta )-S_{j}(\hat{\theta })\right)}^{2}}}{\sigma }$$

where ***N*** is the number of experiments or images, ***θ*** is the (vector) position in the search space, $\hat{\theta }$ is the actual solution (i.e. the true tissue parameters), **S**_*j*_(***θ***) is the signal in the *j*th image for parameters ***θ*** using a particular acquisition scheme, and *σ* represents the notional standard deviation of the noise in the measurement. Therefore, *η* = 1 corresponds to a solution which is one noise standard deviation from the true solution on average over all measurements. Note that no noise was actually added to the simulated signals for this part of the study; setting *σ* allows us to identify good solutions at a particular SNR, and only affects the sharpness of the distributions visualised in section 2.2.2.; it does not otherwise influence any of the results shown.

#### 2.2.1 Signals and solution manifold

Simulated signals for acquisition scheme S1 were calculated for 200 million randomly generated sets of tissue parameters ***θ***, drawn uniformly from initial bound set WPB (no noise was added to these signals). *η* was then evaluated for each of these sets of signals with respect to the actual (although noiseless) signals produced by the model for the ground-truth parameter values, $\hat{\theta }$ in Table 3. For each simulated tissue, the signals with the 1000 lowest residuals (i.e. minimum *η*) were selected.

For the HB data, the manifold containing these top 1000 candidate solutions was visualised using the dimensionality reduction technique of kernel principal component analysis (kPCA) (Mika et al., 1999; van der Maaten et al., 2009). To allow visualisation of the distribution of estimated values of the non-exchange parameters in the presence or absence of exchange, kPCA was performed on the five-dimensional space consisting of *T_1_* and *T_2_* of each pool and MWF, for each of the top 1000 candidate solutions in each scenario.

#### 2.2.2 Search space visualisation

The search space was explored in two different ways. Firstly, 2D projections were created by evaluating *η* on regular grids spanning two selected parameters; for each point on these grids 100,000 random combinations of the other parameters were generated, with *η* evaluated for each and the minimum value stored. Secondly, 2D cuts through the space were made, both in planes intersecting the true solution $\hat{\theta }$, and in planes intersecting solutions found via SRC fitting. For this last case, SRC was performed for data generated for acquisition scheme S1 and S2 (see Supplementary Material results for the latter) and initial bound set B1 with no noise added. In all cases, tissue parameters HB were used (both including and excluding exchange).

### 2.3 Bias and sensitivity of SRC

Monte Carlo simulations were used to investigate the sensitivity of SRC-based mcDESPOT to the initial bound sets. For all Monte Carlo simulations, random Gaussian noise was added to forward modelled signals. The noise level was set to simulate SNR = 100 defined with respect to the mean SPGR signal across all flip angles, so the SNR of individual data points varies as would be the case in reality. In particular, the SNR of the bSSFP scans is greater by up to a factor of 2. In each case, SRC fitting was repeated for data with 1000 different noise realisations. The SNR was chosen given reported in-vivo SNRs from Deoni et al. (76 and 135 in a WM ROI in 14° SPGR and 30° bSSFP images respectively) and to be within the range of SNRs simulated across the mcDESPOT literature (Deoni et al., 2008). Monte Carlo simulations for different SNRs and more detailed results for SNR = 30 (motivated by the ‘high--resolution’ protocol in Bouhrara et al. (Bouhrara and Spencer, 2017)) are shown in Supplementary Material.

#### 2.3.1 Initial bound set sensitivity

All tissue parameter sets (Table 3) were used with acquisition scheme S1, and all initial bound sets (Table 1). In addition, this was repeated for tissue HB (including and excluding exchange) for all acquisition schemes (Table 2). An additional scenario was investigated in which a model that excludes exchange is fitted to signals generated with exchange present. This is motivated by the fact that other researchers have suggested that a stable approach would be to fit a model excluding exchange to in-vivo data (Zhang et al., 2015a; Bouhrara and Spencer, 2016). However, since the literature suggests intercompartmental exchange does exist in-vivo (Dortch et al., 2013; Harkins et al., 2012) we investigated potential bias resulting from the mismatch between model and data. The WML tissue parameter set was used, as the tissue of most interest for our in-vivo results, with *k_FS_* varied from 0s^−1^ (no exchange) to 20s^−1^ in increments of 4s^−1^. For each increment, SRC (with B1-4) was performed for 1000 different noise realisations on data simulated using S1 and with SNR = 100.

#### 2.3.2 Sensitivity to individual parameter changes

To examine the ability of the method to track changes in individual parameters and potential correlations between these, multiple different tissue parameter sets were created by changing one model parameter at a time starting from the HB set. SRC fitting was then used to estimate the parameters, with acquisition scheme S1 and initial bound set B1 assumed.

### 2.4 In-vivo investigation

A whole-brain dataset was acquired for a healthy male volunteer (aged 23 years) using acquisition scheme S1, TE_SPGR_ = 2.25 ms, TE_bSSFP_ = 3.24 ms, field-of-view (FOV) = 230 × 218 × 190 mm^3^, 1.5 × 1.5 × 1.5 mm^3^ resolution on a Philips Achieva 3.0T scanner (Best, Netherlands) with a 32-channel head coil. Since our principal aim was to corroborate our simulation findings, no acceleration was necessary and the acquisition (30 vol) required a scan time of approximately 1 h.

Using the standard deviation approach outlined in Dietrich et al. (2007), we calculated our in-vivo SNR by taking the mean signal across all non-CSF brain regions and all SPGR volumes, and dividing it by the standard deviation of a region of interest outside the head containing only noise, multiplied by the suggested correction factor due to the Rayleigh distribution of noise in magnitude images.

Following recent work on minimising MT effects, the data were acquired using the CSMT method (Teixeira et al., 2018; West et al., 2018). This approach employs non-selective multiband excitation pulses that are designed to have constant total RF power (B_1,rms_ = 1.5 μT) across all flip angles. Hence, the excitation pulses used have 3 bands; the on-resonance band has the flip angle required for excitation, and then two off-resonance bands supply additional RF power to maintain saturation power. Duration of the pulses was 2.5 ms and the off-resonance lobes were located at ±6 kHz. In this work the (on-resonance) flip angles spanned from 2° to 70°; for these cases each off-resonance band had nominal flip angle 49.5° and 0° respectively. The latter was 0° because the 70° pulse produces all saturation on-resonance; the others include off-resonant saturation to maintain the same total power. Please refer to Fig. 2 in Reference 19 for an example illustration RF pulse. SRC was used to process these data with identical parameters to our simulations and using B1-4. For comparison, non-linear least-squares fitting was also performed using the ‘lsqnonlin’ function in MATLAB. We chose the trust-region-reflective algorithm, bounded by B1 and with an initial point defined as the midpoints between the corresponding lower and upper bounds of each parameter. An off-resonance factor was directly fitted for as an additional parameter for in-vivo analysis only, but its inclusion was found to have a negligible effect on search space topology and MWF estimation (data not shown) (Bouhrara et al., 2016).

Although our analysis demonstrated that S1 should give the most precise measurement, in practice, it was found that using all datapoints from S1 caused residual B_0_-artefacts in *T_2F_* and *k_FS_* parameter maps, though MWF appeared relatively unchanged. Signals from the lowest bSSFP0 flip angles were found to be susceptible to off-resonance effects, and hence drifts in the main frequency. Therefore, in our analysis, these datapoints were discarded and this was found to successfully suppress the artefacts with negligible influence on parameter estimation.

## 3 Results

### 3.1 Signal model and search space behaviour

**Fig. 1:**
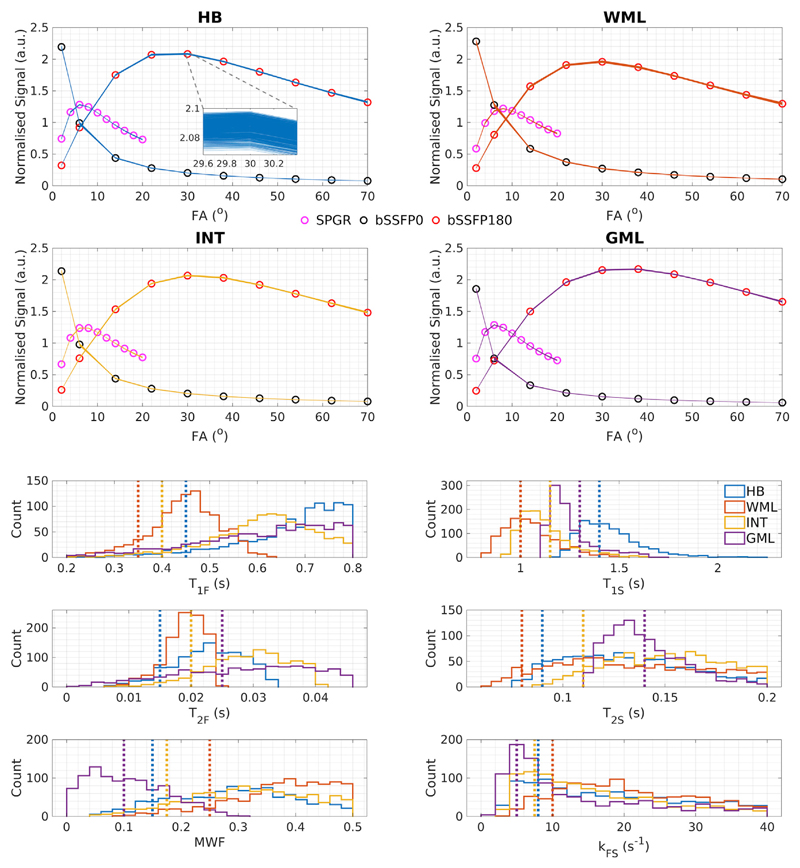
***Top***: Signal profiles for acquisition scheme S1 drawn from 200 million randomly generated combinations of tissue parameters. For each of the tissue types in Table 3, the signal profiles yielding the 1000 lowest residuals from this set were plotted (solid lines) along with the true expected signal profiles for each tissue (circles). In each case, the 1000 different signals are almost exactly coincident so as to be indistinguishable on this diagram. The HB inset (top left) is a zoomed plot of the corresponding bSSFP180 signal profiles to highlight the extent of their degeneracy (note the axis tick label ranges and line thickness); this is also representative of the other signals and different tissue types. ***Bottom***: Histograms of individual tissue parameters for these 1000 lowest residual solutions; vertical dotted lines indicate true parameter values for each tissue type. These histograms indicate that the 1000 lowest residual solutions have a wide spread in underlying parameters, even though their signal profiles are very similar.

Fig. 1 plots the 1000 lowest residual solutions (see 2.2.1) for each of the simulated tissue types, from 200 million randomly generated parameter combinations within the initial bound set WPB. The histograms on the lower half of Fig. 1 plot the distribution of parameter values that these top 1000 solutions span. A very wide range of parameter combinations are present (the histograms are wide), and the mode of values in these histograms do not necessarily correspond to the true parameter values for these tissues (dotted vertical lines). Different tissues create distinct signal curves but within each tissue type all 1000 signals are practically indistinguishable, despite a wide spread of parameter values.

**Fig. 2:**
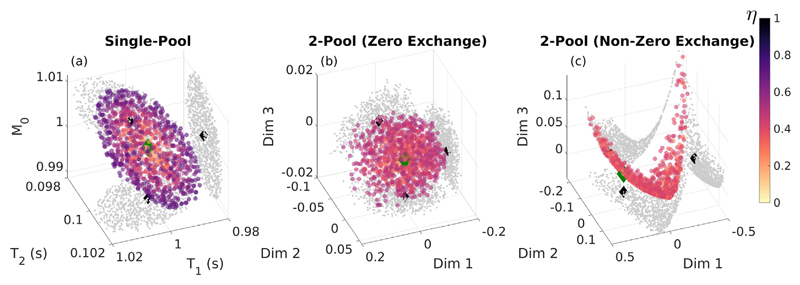
Comparison of the solution manifolds for three cases: (a) single-pool model, (b) two-pool (HB) model excluding exchange and (c) two-pool model (HB) including exchange. Points are coloured according to the value of *η*, the ground-truth is marked as a green diamond and projections onto each axis plane are made.

Fig. 2 presents visualisations of the solution manifold defined by the 1000 lowest residual solutions for tissue type HB in the three scenarios of a single pool (*T_1_* = 1s, *T_2_* = 100 ms, *M_0_* = 1; no dimensionality reduction required), two non-exchanging pools and two pools with exchange. On the figure, the colour is determined by the value of *η*; from this it is clear that the single pool model solutions form a well-defined ellipsoid centred around the true solution (green diamond) with the lowest residual solutions at the centre. The two-pool model *excluding exchange* has a similar manifold of low residual solutions centred around the true solution. However, the two-pool model *including exchange* has a complex topology, with the lowest residual solutions dispersed rather than clustered in one position and the cloud of low residual points is not centred on the true solution.

**Fig. 3:**
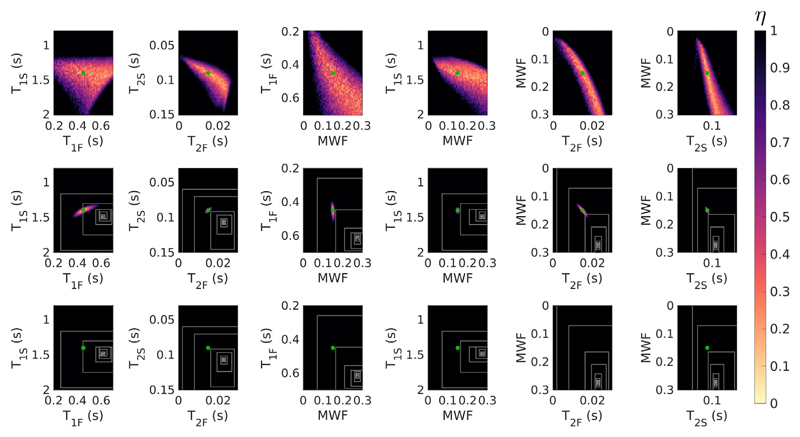
Search space visualisation for tissue HB including exchange and acquisition scheme S1. The colour corresponds to the value of *η* (Eq. (1)). ***Top***: Minimum projection heat maps for parameter pairs. ***Middle***: Cuts of the space through the true solution (green dot). ***Bottom***: Cuts through the solution found by SRC; a low residual region does not appear around the true solution because these cuts are made in a different plane. On the lower two rows, white boxes show the contracting search bounds used by SRC at each iteration. Note that the solution found by SRC is a local minimum of the cost-function though it is not easy to see from the diagram.

**Fig. 4:**
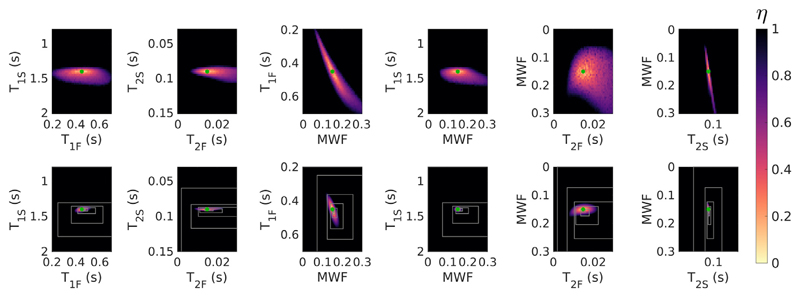
Search space visualisation for tissue HB excluding exchange and using acquisition scheme S1. The colour corresponds to the value of *η* (Eq. (1)). ***Top***: Minimum projection heat maps for parameter pairs. ***Bottom***: Cuts through the true solution, with boxes marking the contracting search bounds used by SRC. Unlike Fig. 3, in this case, SRC converges on the true solution.

The search space for the HB tissue with exchange using acquisition scheme S1 is further visualised in Fig. 3. The top row shows 2D minimum intensity projections of *η* into planes defined by each parameter pairing in turn - it is clear from this that there is a wide spread of parameter combinations with low residuals, and that optimal solution values for different parameters are correlated (particularly the case for MWF with both *T_2F_* and *T_2S_*). The middle row shows a cut (not a projection) through the space at the position of the true solution (green dot) showing that this is indeed a local minimum of the cost-function. However, the white boxes indicate the convergence of the SRC algorithm when used to fit for a signal defined using these parameters; the algorithm converges to a point that appears to have low likelihood. The bottom row however, shows a cut through the space at the position of the SRC solution with the true solution projected into the same planes. It is evident that SRC has found a local minimum, but it is not close to the true solution. Fig. 4 shows equivalent results, now for the same tissue but excluding exchange (i.e. there is no exchange in either the true signal or the fitted signal). The search space still shows correlations between parameters but the SRC method now finds the true solution.

### 3.2 Monte Carlo simulations of SRC

**Fig. 5:**
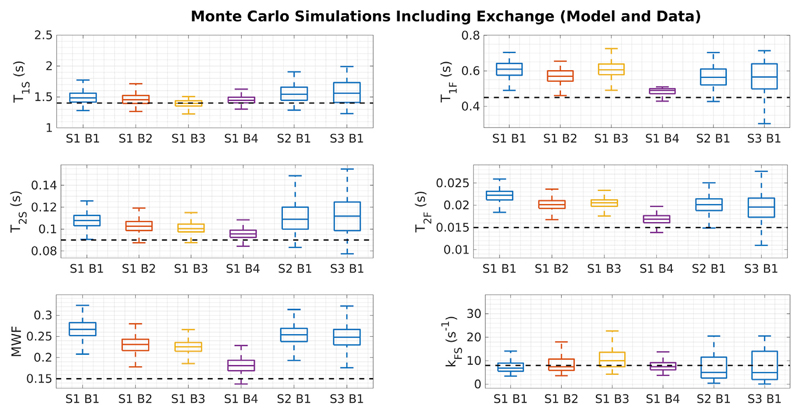
Results of Monte Carlo simulation for SRC fitting for the HB tissue (including exchange). The labels “SxBy” correspond to acquisition scheme *x* and search bounds *y* (Tables 1 and 2). The true parameter values are indicated by the dashed black line. Results indicate estimation biases that are dependent on both the acquisition scheme and initial bound set.

**Fig. 6:**
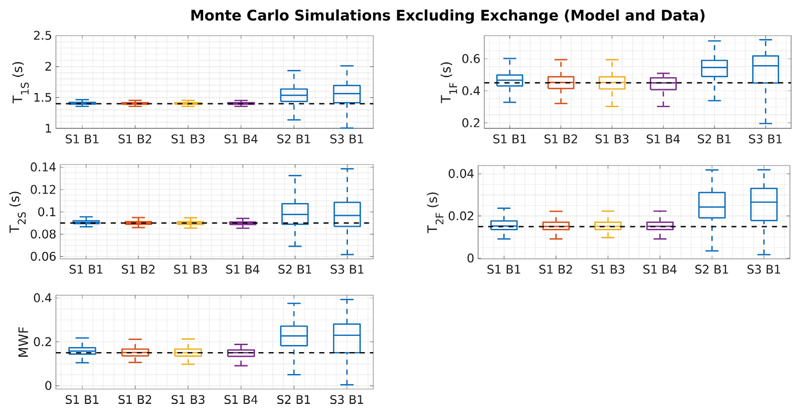
As Fig. 5, but now for the HB tissue excluding exchange between compartments. In this case, there are no large biases in estimation from any combination of acquisition scheme and initial bound set.

Fig. 5 summarises results from Monte Carlo simulation of SRC fitting for tissue HB and for the different acquisition schemes and initial bound sets, with each case repeated over 1000 different noise realisations. The true solution is shown as dotted lines. Each combination of acquisition scheme and bound set leads to different biases on the estimated parameters. For example, using acquisition scheme S1, different initial bound sets lead to consistently differing values of MWF that are all above the true value. Precision (i.e. variance) is also affected by choice of acquisition scheme; S1 is the best, which is to be expected since it comprises the most (30) separate images. Fig. 6 shows the same result for the two pools with no exchange; in this case there are no obvious biases in estimation of any parameter.

**Fig. 7:**
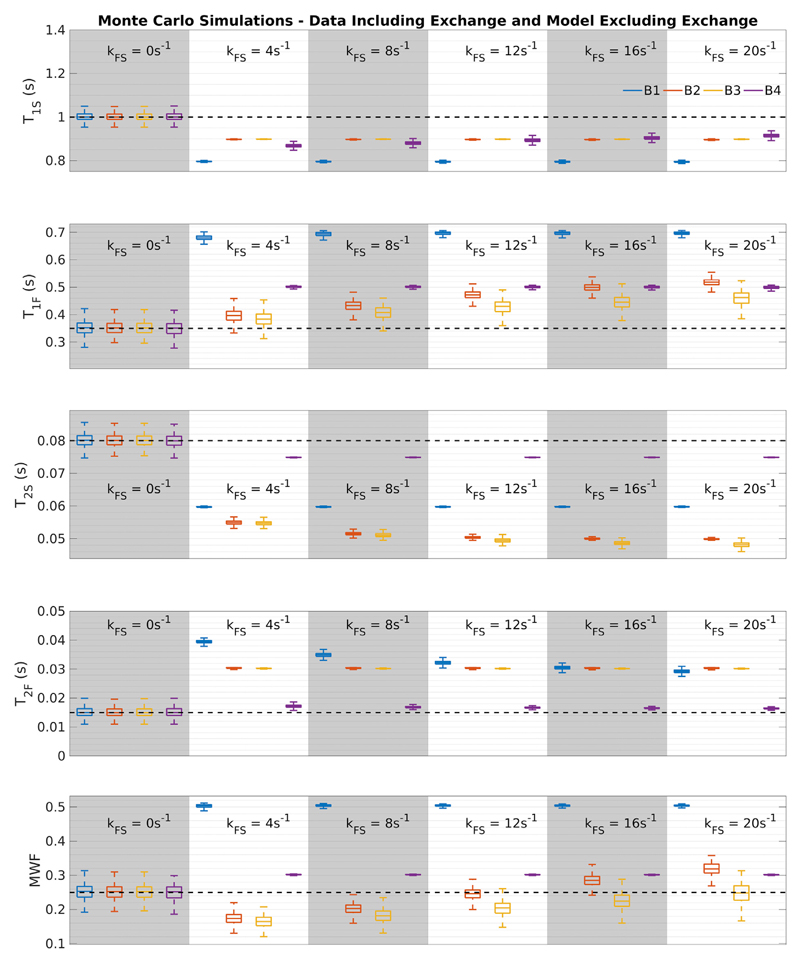
Estimation bias apparent when assuming no exchange with non-zero exchange in underlying simulated data, assuming the WML tissue set. Each box represents SRC parameter estimate from 1000 noise realisations at SNR = 100, and each colour refers to a different initial bound set. The black dashed lines are ground-truth parameter values.

Fig. 7 explores the effect of assuming no exchange during parameter estimation for data generated from forward models that include exchange. Results indicate that a variable degree of bias can result, which in some cases can be quite extreme.

**Fig. 8:**
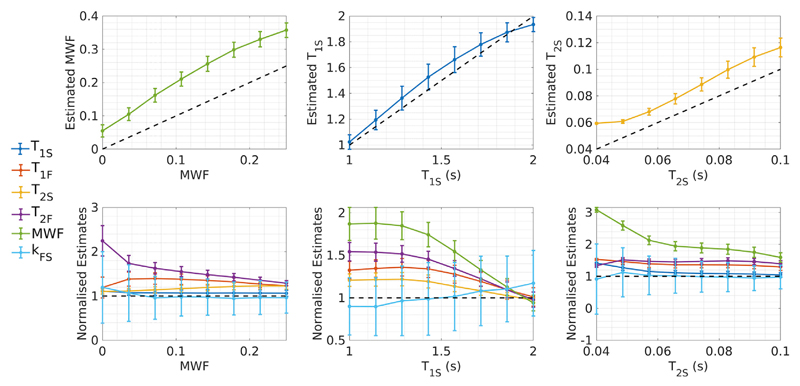
Trends in parameter estimates as ground-truth MWF, *T_1S_* and *T_2S_* are incremented, assuming acquisition scheme S1, bound set B1 and SNR = 100. All other ground-truth parameter values for each trial were as per tissue set HB (including exchange). The top row shows the change in each estimated parameter as it is itself varied - estimated parameters increase monotonically with the true values but not always linearly. The bottom row shows the changes in all other parameters as one is varied, in each case normalised to the true value. For example, if *T_2S_* is reduced to 40 ms, the estimated MWF will increase to three times the true value, when it was actually unchanged in the underlying model. Note that due to the sensitivity of SRC to acquisition scheme and fitting bound set, these plots are only true for S1 and B1.

Fig. 8 summarises the results of Monte Carlo simulations performed by individually incrementing ground-truth values for MWF, *T_1S_* and *T_2S_* to create twenty-four unique tissue sets for data simulation. The results indicate that while increases in MWF, *T_1S_* and *T_2S_* all lead to monotonic increases in their estimates, there is an offset and there is some nonlinearity in the response. Furthermore, the second row of the figure shows how each of the other parameters varies in each case - here we see that if the true underlying *T_1S_* or *T_2S_* change, this may also result in a large change in the estimated MWF, even though in those cases MWF has actually remained fixed.

### 3.3 SRC repeatability: simulations and in-vivo study

**Fig. 9:**
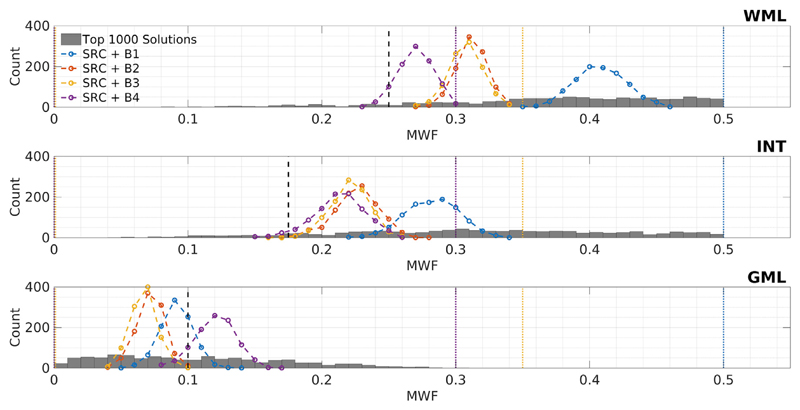
Summary of Monte Carlo simulation of SRC fitting of a model including exchange and using different search bounds. Each panel corresponds to a different tissue type and focuses on MWF (though all parameters were estimated). Since HB is a mixture of the other three tissue types (as the low residual solutions in Fig. 1 shows), it is excluded from this analysis for clarity. Ground-truth values are shown as black dashed lines, histograms of SRC estimated MWF by coloured dotted lines and bounds by colour-matched vertical lines. Grey bars correspond to the low residual solutions identified in Fig. 1. Similar plots are shown in Supplementary Material for when a model excluding exchange is fitted to WML, INT and GML data.

The high degree of degeneracy in the two-pool model with exchange (Fig. 1) suggests that in-vivo parameter maps should have poor precision, yet many studies have shown that this is not the case (Deoni et al., 2013; Zhang et al., 2015b; Kolind and Deoni, 2011). This is illustrated by Fig. 9, which collates the result of SRC fitting for each uniquely defined tissue type (WML, INT, GML), using acquisition scheme S1, SNR = 100 and varying initial bound sets. The grey bars represent the low residual solutions plotted on Fig. 1, whereas the coloured lines plot the histograms of the MWF found from SRC estimation with each initial bound set. It is apparent that SRC results in a smaller than expected variance but a variable degree of bias that depends on the initial bound set used. Each different set would therefore yield plausible and repeatable measures that are different to the results that would be obtained by using different search bounds. Supporting Figure SF6 shows equivalent results for the no exchange case, where the distribution of MWF values estimated by SRC more closely resembles the distribution of low residual solutions within the search bounds. That figure also shows results for the case in which the forward model includes exchange, but this is excluded from the fitting; results are highly unpredictable and far from the true values.

**Fig. 10:**
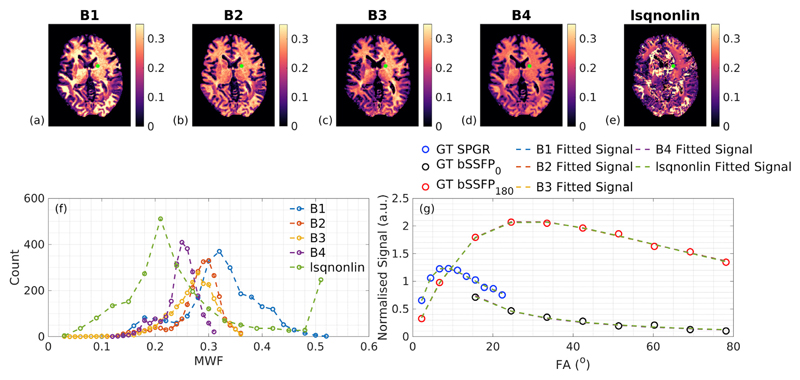
In-vivo MWF maps calculated using SRC with different bounds and standard non-linear fitting (top). Our SNR, calculated as described in the methods, was approximately 120, which is in-line with our simulations. SRC produces plausible contrast but the absolute level of MWF depends on the bounds, whereas non-linear fitting often converges to highly different minima. Corresponding histograms from WM show clear similarities are seen between the bound-sensitivity shown here and in Fig. 9. The bottom right tile shows data (circles) acquired from a single internal capsule voxel (marked in green in top panel) and the signal curves corresponding to the parameters for each fitting method/bound set (dashed lines), which are almost indistinguishable despite the very different levels of MWF. This implies that the different fitted parameter sets fit the data equally well.

Finally, Fig. 10 shows in-vivo MWF estimated maps (acquisition scheme S1) fitted using the different initial bound sets. Each map (a-d), except for ‘lsqnonlin’ (e) which was produced by classic non-linear optimisation, shows plausible GM-WM contrast; however, the absolute values are variable. Histograms from a WM mask (acquired using FSL BET and FAST tools (Jenkinson et al., 2012)) show different biases in the estimated MWF, depending on the initial bound set, similar to Fig. 9. Representative signal plots from an individual voxel show that all solutions pass equally well through the measured data points, even though the fitted parameters are rather different. Supporting Figure SF5 shows equivalent results for the same data, but this time fitted assuming *k_FS_* = 0. Here, a much wider spread of MWF values results, in each case clustering close to the upper bound of the initial search parameter.

## 4 Discussion

This work investigated sources of parameter estimation bias from the SRC fitting method for mcDESPOT data. This was motivated by previous theoretical analyses (Lankford and Does, 2013) suggesting that mcDESPOT should be unable to provide useful parameter estimates at attainable SNR. Nevertheless, there are many examples in the literature of plausible mcDESPOT MWF maps with precision in excess of what would be implied by the Cramer-Rao Lower Bound (Lankford and Does, 2013), suggestive of a biased fitting. It has also been shown by others that removal of exchange results in a more stable model (Bouhrara and Spencer, 2016). However, inclusion of exchange is biologically plausible and likely carries important microstructural information (Harkins et al., 2012). Studies have observed exchange in ex-vivo rat optic and frog sciatic nerve (Dortch et al., 2013), and a recent 7T study found evidence for a relatively fast exchange in human brain (van Gelderen and Duyn, 2018). Hence, assuming no exchange may also be expected to cause some estimation bias.

The challenge for mcDESPOT parameter estimation in the presence of intercompartment exchange is clearly illustrated by Fig. 1, which shows that the fitting problem is highly degenerate. For each tissue type we have plotted 1000 signal curves that cover a wide spread in underlying parameters but are indistinguishable by eye. Fig. 2 demonstrates that these degenerate solutions form a complex hypersurface in the search space. The result of this is that SRC fitting converges to local minimum of the cost-function (Fig. 3) but there is no way to determine which is the true solution. The result for parameter estimation is then illustrated by Fig. 5: Monte Carlo analysis demonstrates that there is an unpredictable degree of bias in the parameter values, and that this bias is a function of both the acquisition scheme (i.e. which combinations of flip angles, repetition times, and balanced or spoiled sequences are used) and the initial bound sets used by the SRC algorithm.

When exchange is not present, the solutions are much better behaved (Fig. 2), as also shown by Bouhrara et al. (Bouhrara and Spencer, 2016) This is also borne out by Monte Carlo simulations (Fig. 6) using different acquisition schemes and initial bound sets. Though precision varies between different choices of acquisition scheme, as would be expected since they include different numbers of measurements, all combinations correctly estimate parameter values within one standard deviation of the true values. As another illustration, the example search space in Fig. 4 contains a well-defined cost-function minimum that coincides with the true solution, and this is reliably found by the SRC fitting. The case of two exchanging pools has a very different search space with many equally likely solutions that have widely differing parameter values. This provides a mechanism for the observed bias through interaction between the complex manifold of degenerate solutions and the progressively contracting search bounds employed by SRC. Different choices of initial bound set largely dictate where in this degenerate manifold the algorithm will converge. Hence, the observed variance in estimation when using SRC is expected to be smaller than the true spread of possible degenerate solutions. This was confirmed by Monte Carlo simulation using different initial bound sets (see Fig. 9).

Results from our in-vivo investigation are in agreement with these simulations. Fig. 10 shows four different MWF maps reconstructed from the same underlying data, with the same SRC algorithm except that the initial bound sets are different. The result is four maps that are all different but appear reasonable. The histograms of estimated MWF in WM show qualitatively similar behaviour to Fig. 9. Signal profiles from a single voxel, also shown in Fig. 10, indicate that the solutions found are degenerate even though the estimated parameters are different (see Table 4). This sensitivity to search bounds has been commented on in previous studies, for example Zhang et al. (2015a) demonstrated significant differences in MWF when extending the search bounds. Note that the issue is not that the true solution lies outside of the defined bounds, such that the optimiser hits these bounds when converging. All of the defined initial bound sets in Table 1 contain all of the modelled tissue ‘true’ values in Table 3, for example. Rather, we conclude from this investigation that the different bounds interact with the hypersurface of degenerate solutions, leading to a different subset being found, depending on the initial bound set. A related problem is highlighted by Fig. 8: though this does show that monotonic changes in underlying parameters also lead to monotonic changes in their estimates, these relationships aren't always linear and more problematically, they can be coupled with marked changes in the estimated values of other parameters that aren't actually changing. A clear example is that changes in *T_1S_* and *T_2S_* both lead to changes in estimated MWF. As a comparison with SRC, a standard non-linear fitting algorithm was also used (‘lsqnonlin’, see Fig. 10) and did not give an anatomically plausible MWF. This approach yielded ‘noisy’ looking parameter estimates, since it did not force convergence onto a smaller subset of the possible solutions in the way that SRC does.

One strategy to avoid the poor behaviour of the model with exchange is to exclude it from parameter estimation (Bouhrara and Spencer, 2016; Bouhrara et al., 2016). In this case, an important question is how does the presence of exchange in the actual data manifest in parameter estimation? To investigate this, we generated using variable non-zero *k_FS_* and then fitted it with *k_FS_* fixed at 0s^−1^. The results (Fig. 7) suggest that the derived parameter estimates would be highly variable depending on the actual value of *k_FS_*. This was also observed with the in-vivo dataset (Supporting Figure SF5); fitting a model excluding exchange led to MWF estimates that also approach or hit the upper bounds of the search space for each initial bound set used. Slight deviations in MWF estimates between simulations and in-vivo are due to differences between model parameter values. The Bayesian method proposed by Bouhrara et al. (Bouhrara and Spencer, 2016) may provide a means to better constrain the ‘excluding exchange’ model, but we note that the authors of that study did not report how the actual presence of exchange might affect their results.

### 4.1 Magnetisation transfer effects

The signal models used in this work excluded a semisolid proton pool that would lead to MT effects, instead the focus of this investigation was the inherent ability with which a two-pool model including exchange could be used for parameter estimation. Clearly a related issue is that MT effects may also affect the estimation in-vivo (Zhang et al., 2015b), hence we used the CSMT approach which leads to significantly improved stability over the normal approach to VFA imaging (Teixeira et al., 2018). A key insight is that using the CSMT approach makes the two-pool system consisting of one MR visible (water) proton pool and one invisible semisolid pool behave as a single-pool system. In recent work we have extended this to show that a system with two visible water pools (e.g. myelin-water and IE-water) and one semisolid pool, will behave as a system with two visible pools only (i.e. the model used in this work) under the same CSMT conditions (West et al., 2018). However, the issues with model degeneracy and SRC fitting were found to dominate over the MT effects, and hence they are the main subject of this paper. Fig. 10 shows that the acquired CSMT signals are well-described by the standard two-pool model.

### 4.2 Other sources of bias in data

The majority of the results presented (apart from the in-vivo data) investigate a ‘best case scenario’ in which the data and model fitting use the same two-pool model. However, there are limitations with how well this model can describe in-vivo data. For example, Deoni et al. have proposed the inclusion of more than two pools of water protons to avoid partial volume effects with CSF (Deoni et al., 2013). Though beneficial for in-vivo analysis of voxels on the edge of the parenchyma, the inclusion of additional model parameters would likely lead to even further degeneracy and hence this was not investigated here.

On the subject of MT effects, the CSMT approach is designed to force the system to behave as if the semisolid pool is not present; other implementations of mcDESPOT would differ from this, and hence there may also be systematic deviations present in other data that aren't present in ours. Similarly, it is well known that RF spoiling (for the SPGR sequences) is not perfect, and some have suggested corrections for this (Preibisch and Deichmann, 2009); this was neglected in this work, as it appears to be throughout the literature on mcDESPOT. Systematic deviations between model and tissue are a source of bias that are not present in our simulation analysis. However, by examining the way in which a two-pool model consisting of IE and myelin water can be fitted to itself, we have demonstrated that even if the data were to fit the model perfectly, biases in fitting would dominate. Additionally, the biases found in in-vivo data appear to be consistent with the ‘best case’ simulation study.

## 5 Conclusions

In conclusion, our results indicate that mcDESPOT using a signal model that includes intercompartmental exchange does not provide objective estimates of the underlying model parameters. Although stable and reproducible results can be obtained, the degeneracy of the model means that the parameter values obtained (focusing particularly on MWF) are functions of both the particular acquisition scheme used and the algorithm used to estimate the tissue parameters, including specific parameters of that fitting approach. Hence, it does not make sense to compare MWF estimated by mcDESPOT between studies unless the exact same acquisition scheme and fitting algorithm are used with the same search parameters. Full transparency in methods is required to ensure this. If these conditions are met, then the approach could potentially be used as a ‘semi-quantitative’ measure. However, even in this case, caution is required since changes in some tissue parameters can erroneously appear as changes in others. As also found by others, if exchange can be excluded then it is possible to make more objective measurements, however if exchange is neglected during fitting for a system in which intercompartmental exchange is actually present, unpredictable parameter bias may still result.

## Supplementary Material

Supplementary data to this article can be found online at https://doi.org/10.1016/j.neuroimage.2019.03.049.

Supplementary Material

## Figures and Tables

**Table 1:** Different initial bound sets tested for Monte Carlo simulations. B1 is used by Bouhrara et al. (‘restricted bounds’ with extra *k_FS_* limits), B2 by Deoni et al. (‘default boundary conditions’), B3 is from an in-house study and B4 is used by Zhang et al. The bottom row is a set with the widest parameter bounds (WPB) possible from B1-4.

Bound Set	Literature Reference	*T_1F_* (s)		*T_1S_* (s)		*T_2F_* (ms)		*T_2S_* (ms)		MWF		*k_FS_* (s^−1^)	
		LB	UB	LB	UB	LB	UB	LB	UB	LB	UB	LB	UB
B1	Bouhrara et al. (2016)	0.2	0.7	0.8	2	2	40	60	160	0	0.5	0.5	20
B2	Deoni and Kolind (2015)	0.3	0.65	0.9	5	1	30	50	165	0	0.35	1.67	40
B3	N/A	0.3	0.8	0.9	1.5	10	30	40	150	0.001	0.35	1.67	40
B4	Zhang et al. (2015a)	0.2	0.5	0.7	2.5	2	45	75	200	1e-7	0.3	0.5	20
WPB	N/A	0.2	0.8	0.7	5	1	45	40	200	0	0.5	0.5	40

**Table 2:** Different acquisition schemes tested for Monte Carlo simulations. S1 is from Bouhrara and Spencer, S2 is from Deoni et al. (defined as ‘simulated acquisition parameters’) and S3 is an example reduced scheme used in-house for single-pool relaxometry. bSSFP180 is the usual phase-cycled bSSFP sequence and bSSFP0 is a non-phase cycled version.

Acquisition Scheme	Literature Reference	TR_SPGR_ (ms)	TR_bSSFP_ (ms)	FA_SPGR_ (°)	FA_bSSFP180_ (°)	FA_bSSFP0_ (°)
S1	Bouhrara and Spencer (2016)	6.5	6.5	2, 4, 6, 8, 10, 12, 14, 16, 18, 20	2, 6, 14, 22, 30, 38, 46, 54, 62, 70	2, 6, 14, 22, 30, 38, 46, 54, 62, 70
S2	Deoni and Kolind (2015)	5.6	4.4	4, 5, 6, 7, 9, 11, 14, 18	12, 16, 19, 23, 27, 34, 50, 70	12, 16, 19, 23, 27, 34, 50, 70
S3	N/A	7.0	7.0	6, 8, 10, 12, 14, 16	15, 25, 35, 45, 55, 65	25, 55

**Table 3:** Tissue parameter sets used in this work and chosen to be within all bound sets in Table 1 (see text for more details). *Exchange is either included at 8s^−1^ or excluded (0s^−1^) for the HB model. On-resonance is assumed for each set and so, fitting of an off-resonance factor is not required.

Tissue Set	*T_1F_* (s)	*T_1S_* (s)	*T_2F_* (ms)	*T_2S_* (ms)	MWF	*k_FS_* (s^−1^)
HB	0.45	1.4	15	90	0.15	8/0*
WML	0.35	1.0	15	80	0.25	10
INT	0.4	1.15	20	110	0.175	7.5
GML	0.45	1.3	25	140	0.1	5

**Table 4:** Estimated parameters from each fitting attempt, for the WM voxel marked in Fig. 10.

	*T_1F_* (s)	*T_1S_* (s)	*T_2F_* (ms)	*T_2S_* (ms)	MWF	*k_FS_* (s^−1^)
B1	0.472	0.901	15.7	105	0.373	5.04
B2	0.397	0.943	12.9	106	0.297	8.38
B3	0.411	0.945	13.4	102	0.317	6.07
B4	0.343	1.01	10.9	98.3	0.268	5.93
lsqnonlin (B1)	0.585	0.800	18.8	93.1	0.500	1.29
