# Supplementary material for: Inherent and unpredictable bias in multi-component DESPOT myelin water fraction estimation
Source: Neuroimage. Author manuscript; Available in PMC 2020 Mar 27. (PMC7100802; doi:10.1016/j.neuroimage.2019.03.049)
Supplement: Supplementary Material [file EMS85964-supplement-Supplementary_Material.docx]

**Supplementary Material: SRC Performance**

Monte Carlo simulations were run assuming the HB tissue set, acquisition scheme S1 and initial bound set B1, 1000 noise realisations and different SNR levels for a model that includes exchange. SF1 shows that whereas the standard deviations of parameter estimate distributions decrease as SNR increases for each model parameter, their mean, and therefore bias with respect to their ground-truth values, is consistent.


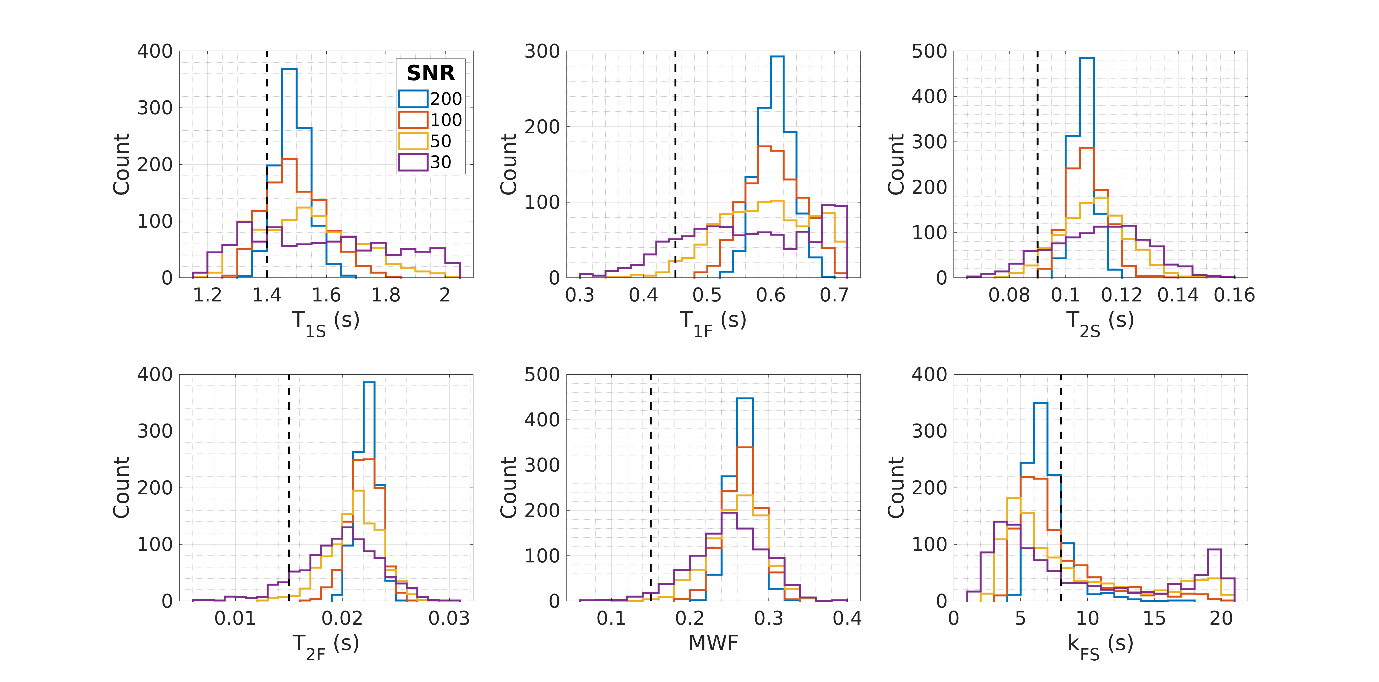


SF1: Parameter estimate histograms for Monte Carlo simulations, assuming SNR = 30, 50, 100 and 200. An SNR = 100 was chosen for our simulations because it yields an approximately Gaussian distribution for all model parameters (besides the consistently unstable *k_FS_*) and is clinically-achievable. For a high-resolution mcDESPOT protocol, SNR = 30 might be more appropriate; example simulation results with this SNR are shown below.

To represent a higher-resolution protocol, as suggested by Bouhrara *et al.*^22^ all Monte Carlo simulations were repeated assuming SNR = 30. Below are equivalent versions of Figures 5 and 6 for this SNR.

**
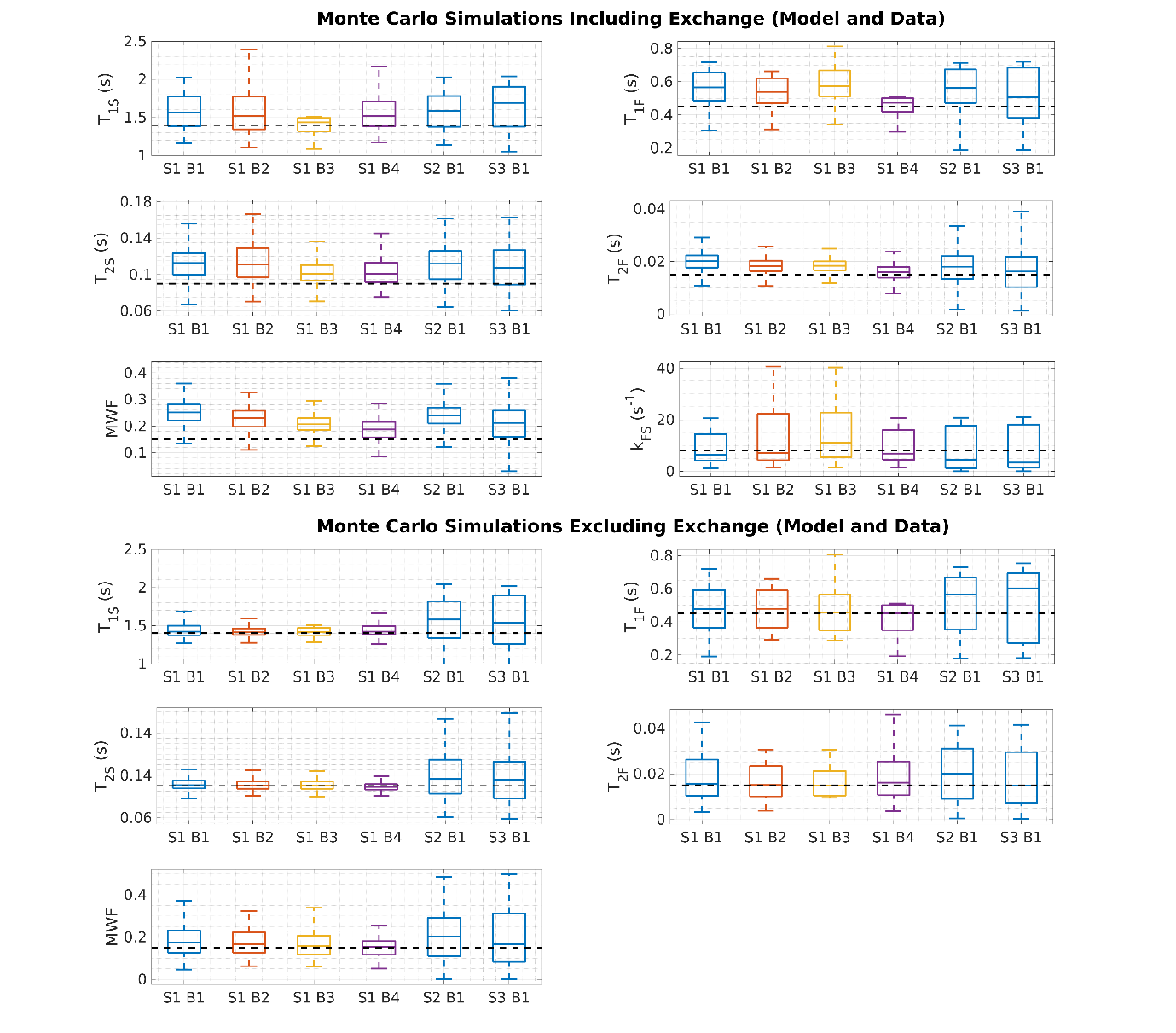
**

SF2: Results of Monte Carlo simulation for SRC fitting for the HB tissue (including and excluding exchange), assuming SNR = 30. Results when exchange is included indicate estimation biases that are dependent on both the acquisition scheme and initial bound set. When exchange is excluded from the model, no large biases exist in estimation from any combination of acquisition scheme and initial bound set. Despite a significantly lower SNR, the same trends are apparent here as in Figures 5 and 6, the only difference being a slightly increased standard deviation of parameter estimates in most cases, as expected. Note, all trends apparent in Figures 2-9 do not change significantly when the simulated SNR is modified.

**Supplementary Material: Search Space and Degeneracy Analysis**

Following the methods outlined in *2.2.2. Search Space Visualisation*, Figures 3 and 4 are replicated but with ground-truth data simulated using acquisition scheme S2 (instead of S1).


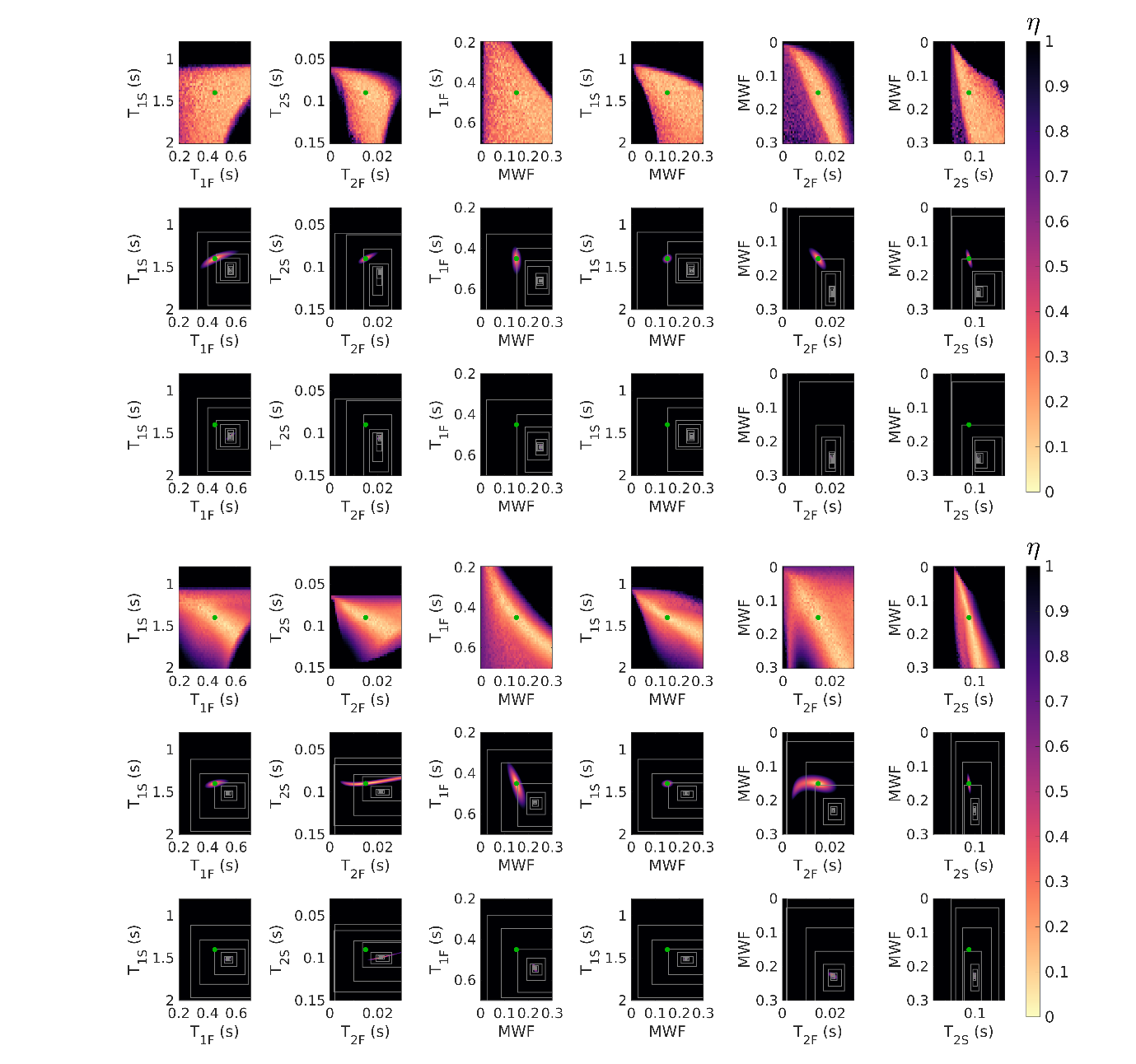


SF3: Search space analysis for including exchange (upper half) and excluding exchange (lower half), assuming acquisition scheme S2. Format is equivalent to Figures 3 and 4. For the latter, SRC now fails to locate the ground-truth exactly and the search space has a much-increased degeneracy compared to Figure 4. The distribution of low residual solutions and parameter estimates obtained by SRC are mostly unaffected by the inclusion or exclusion of exchange when this reduced acquisition scheme is used.

To further highlight the degeneracy of the mcDESPOT search space, below are minimum projection maps of normalised residuals for the case of fitting a single-pool model to itself (e.g. DESPOT1/2-like), assuming on-resonance and produced using an identical strategy to those presented in Figures 3 and 4 and SF3. In each subplot, a more well-defined cost-function minimum exists, centred on the ground-truth point.

**
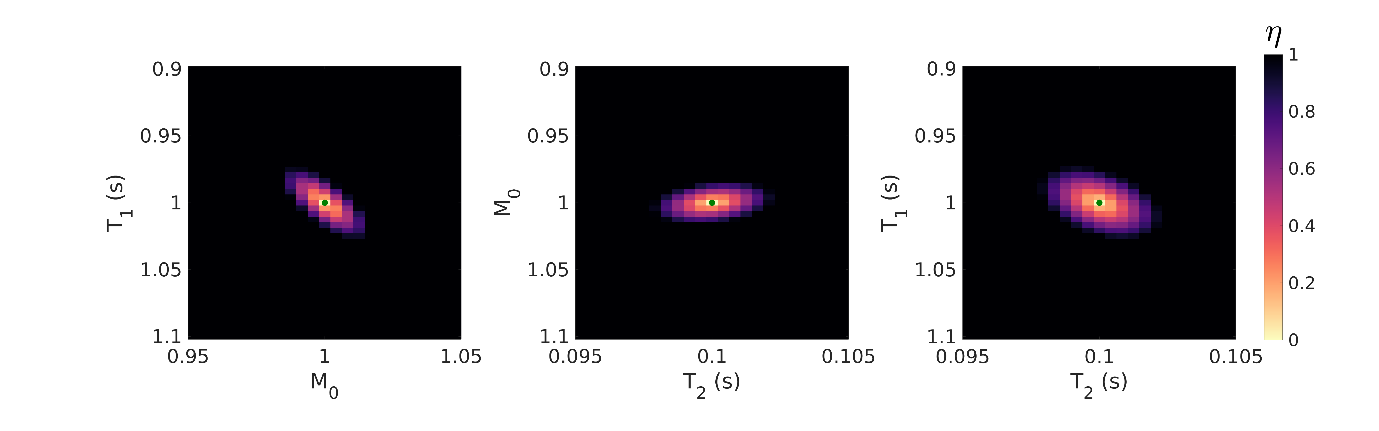
**

SF4: Single-pool minimum projection normalised residual maps for all possible parameter pair combinations. The same tissue parameters as in Figure 2 were used. Green dots indicate the ground-truth values.

SF5 shows MWF maps calculated following fitting of a model excluding exchange to the in-vivo data acquired using acquisition scheme S1 but using different initial bound sets. These can thus be directly compared to those in Figure 10. The bottom panel shows the distribution of MWF values in a WM mask.


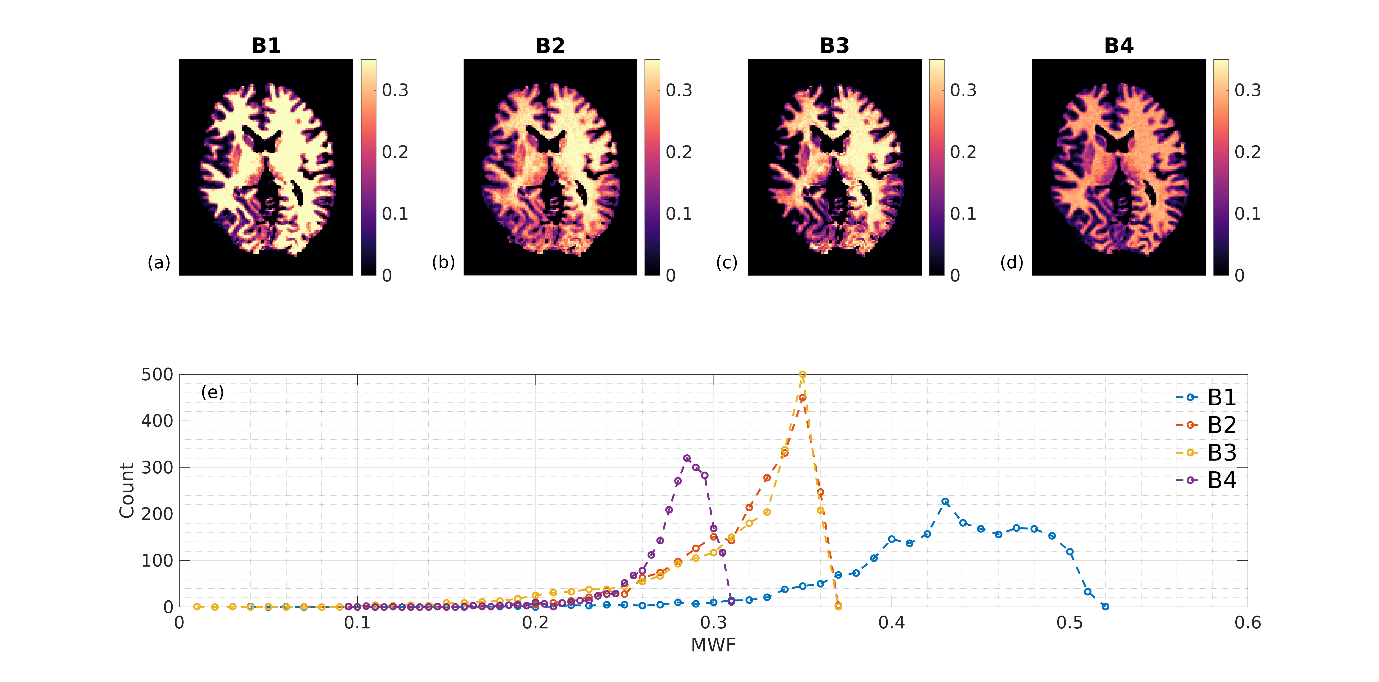


SF5: Resulting MWF maps and WM MWF values generated when fitting a model excluding exchange to the acquired in-vivo data. We see accumulation of MWF estimates at corresponding upper bounds for each initial bound set used. These trends are somewhat corroborated by the simulated bound-sensitivity results shown in the lower tile of SF6, though some differences are expected since a less representative model is fitted to the data.

SF6 shows equivalent plots to Figure 9 but when a model excluding exchange is fitted to WML, INT and GML simulated data that either excludes (upper tile) or includes (lower tile) exchange. Once again, the low residual solutions are shown in the background, with Monte Carlo SRC results overlaid.

**
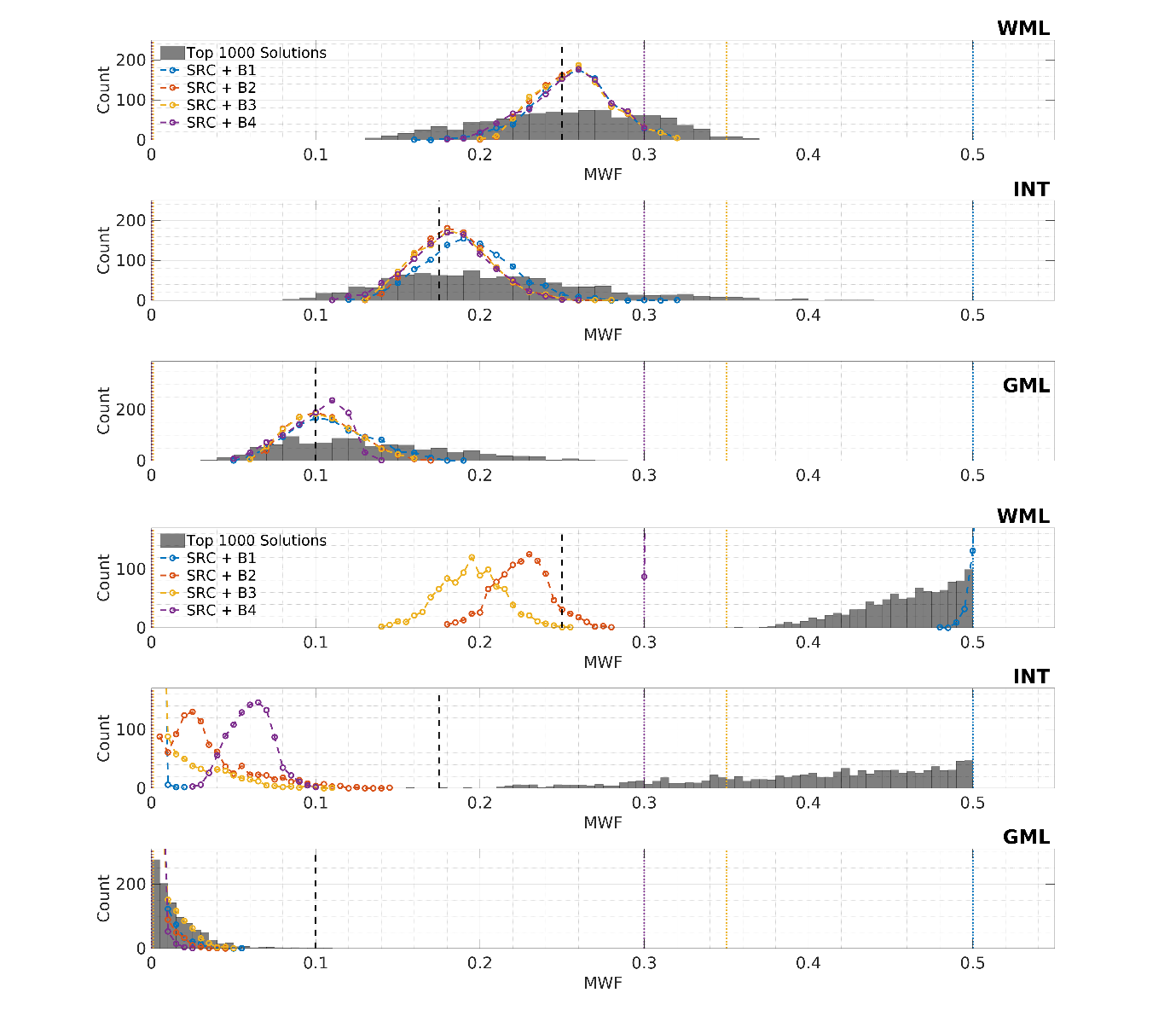
**

SF6: ***Top***: Summary of Monte Carlo simulation of SRC fitting of a model excluding exchange (using different search bounds) to simulated data that also excludes exchange. Bound limits and ground-truth values are marked as in Figure 9. Lowest residual solutions are more centred on their corresponding ground-truth values and an improved inter-bound consistency is evident compared to when exchange exists and is fitted for (Figure 9). ***Bottom***: An equivalent plot for fitting a model excluding exchange to ‘standard’ WML, INT and GML simulated data that includes exchange (at rates listed in Table 3). For all tissue types, the lowest residual solutions are removed from the ground-truth and SRC inconsistently samples the search space, with some parameter estimates noticeably aggregating at their respective lower or upper bounds.

**Supplementary Material: Effect of Low bSSFP0 Flip Angles**

As discussed in Section 2.4., low flip angle bSSFP0 images were excluded from our in-vivo analysis due to the presence of B_0_-artefacts in the reconstructed parameter maps. Shown below are all maps obtained following SRC fitting (with bound set B1) of all in-vivo data acquired using acquisition scheme S1 (i.e. the same data analysed in Figure 10 and SF5 but now including the previously excluded low flip angle bSSFP0 measurements). Clear B_0_-artefacts affect parameter estimation, especially *T_2F_* and *k_FS_*, whilst location-dependent underestimation of MWF is apparent compared to maps in Figure 10.


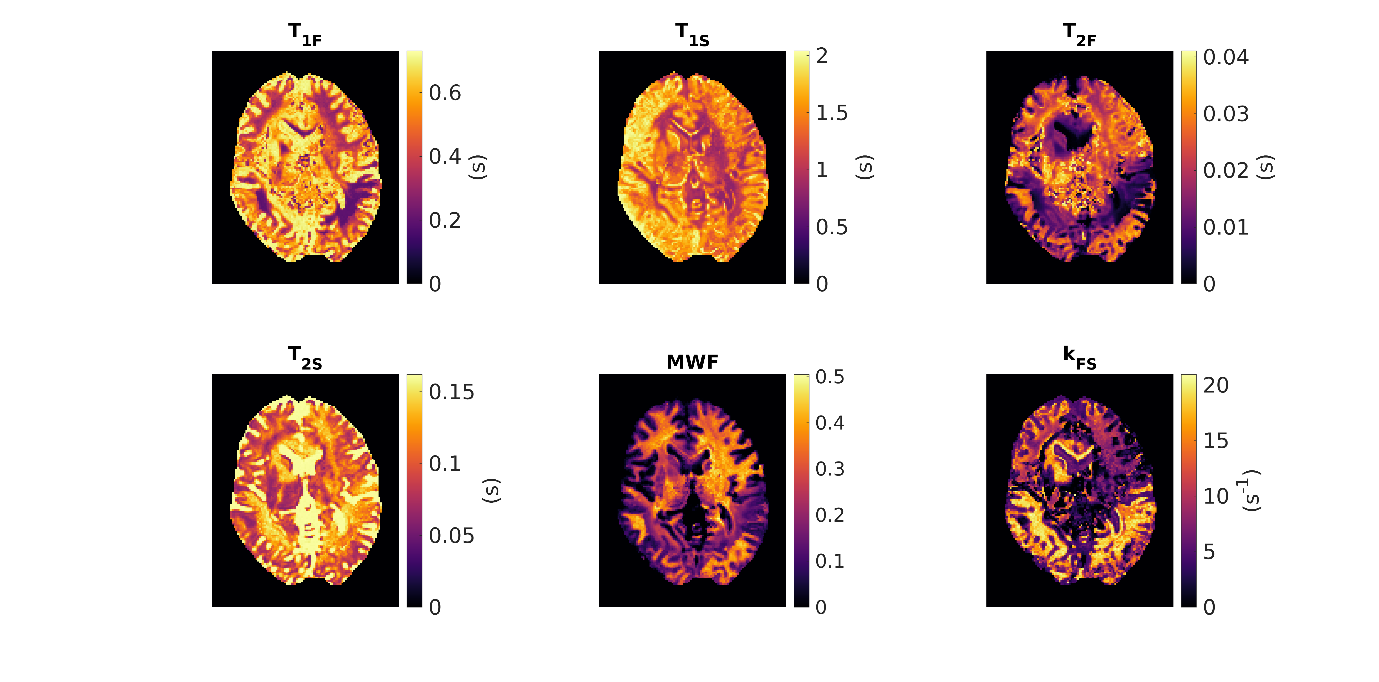


SF7: Parameter maps obtained when including the FA = 2˚ and FA = 6˚ bSSFP0 images for in-vivo data analysis. Parameter estimation is clearly affected by B_0_-artefacts that arise due to the instability of these datapoints. Most importantly, MWF values appear partially underestimated and so this motivates the exclusion of these low flip angle datapoints from in-vivo mcDESPOT analysis.
